# SARS-CoV-2 B.1.1.529 (Omicron) Variant — United States, December 1–8, 2021

**DOI:** 10.15585/mmwr.mm7050e1

**Published:** 2021-12-17

**Authors:** CDC COVID-19 Response Team

A new variant of SARS-CoV-2 (the virus that causes COVID-19), B.1.1.529 (Omicron) (*1*), was first reported to the World Health Organization (WHO) by South Africa on November 24, 2021. Omicron has numerous mutations with potential to increase transmissibility, confer resistance to therapeutics, or partially escape infection- or vaccine-induced immunity (*2*). On November 26, WHO designated B.1.1.529 as a variant of concern (*3*), as did the U.S. SARS-CoV-2 Interagency Group (SIG)* on November 30. On December 1, the first case of COVID-19 attributed to the Omicron variant was reported in the United States. As of December 8, a total of 22 states had identified at least one Omicron variant case, including some that indicate community transmission. Among 43 cases with initial follow-up, one hospitalization and no deaths were reported. This report summarizes U.S. surveillance for SARS-CoV-2 variants, characteristics of the initial persons investigated with COVID-19 attributed to the Omicron variant and public health measures implemented to slow the spread of Omicron in the United States. Implementation of concurrent prevention strategies, including vaccination, masking, increasing ventilation, testing, quarantine, and isolation, are recommended to slow transmission of SARS-CoV-2, including variants such as Omicron, and to protect against severe illness and death from COVID-19.

## Surveillance for SARS-CoV-2 Variants and Initial Detection of Omicron in the United States

CDC has a multifaceted surveillance system for analyzing SARS-CoV-2 variants circulating in the United States. This system obtains genomic surveillance data from 1) National SARS-CoV-2 Strain Surveillance, 2) CDC-supported contracts with several commercial diagnostic laboratories, and 3) public repositories (the Global Initiative on Sharing Avian Influenza Data [GISAID]^†^ and the National Center for Biotechnology Information [NCBI]^§^) of randomly sampled viruses with metadata tagging of sequences by various partners. Genomic surveillance is implemented in partnership with state and local public health laboratories, the Association of Public Health Laboratories, and other academic and government partners.^¶^ As of the week ending December 4, the SARS-CoV-2 B.1.617.2 (Delta) variant was estimated to account for 99.9% of SARS-CoV-2 circulating in the United States.** Based on CDC analysis of the sequences currently available, and accounting for clustering, CDC estimates a 95% chance of detecting the Omicron variant if it accounted for ≥0.03% of circulating SARS-CoV-2 lineages during the week ending November 13 and for ≥0.05% of circulating lineages during the week ending November 20 (*4*).

**FIGURE:**
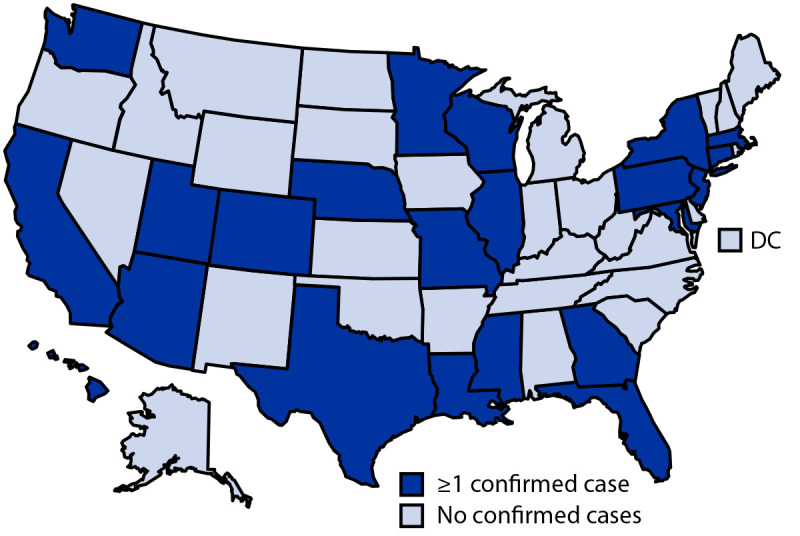
States reporting at least one confirmed SARS-CoV-2 B.1.1.529 (Omicron) variant COVID-19 case — United States, December 1–8, 2021 **Abbreviation:** DC = District of Columbia.

To accelerate detection of COVID-19 cases attributed to the Omicron variant until they are common enough to be reliably measured by routine genomic surveillance, enhanced surveillance was initiated through National SARS-CoV-2 Strain Surveillance on November 28. The method is based on rapid screening for S-gene target failures (SGTFs) by polymerase chain reaction (PCR)–based diagnostic assays to flag potential cases of Omicron variant infection for confirmation by genomic sequencing (*5*). Specimens that display SGTFs have a higher likelihood to be Omicron (although SGTFs are not unique to Omicron) based on a mutation (69–70 deletion) that reduces S-gene target amplification in some PCR assays.

The first U.S. case of COVID-19 attributed to the Omicron variant was identified on December 1. As of December 8, cases had been reported from across the country; 22 states have reported at least one case (Arizona, California, Colorado, Connecticut, Florida, Georgia, Hawaii, Illinois, Louisiana, Maryland, Massachusetts, Minnesota, Mississippi, Missouri, Nebraska, New Jersey, New York, Pennsylvania, Texas, Utah, Washington, and Wisconsin) (Figure). This activity was reviewed by CDC and was conducted consistent with applicable federal law and CDC policy.^††^

## Characteristics of the First Investigated U.S. COVID-19 Cases Attributed to the Omicron Variant

Details are available for 43 cases of COVID-19 attributed to the Omicron variant; 25 (58%) were in persons aged 18–39 years (Table). The earliest date of symptom onset was November 15 in a person with a history of international travel. Fourteen (33%) persons reported international travel during the 14 days preceding symptom onset or receipt of a positive test result. Among these cases of COVID-19 attributed to the Omicron variant, 34 (79%) occurred in persons who completed the primary series of an FDA-authorized or approved COVID-19 vaccine ≥14 days before symptom onset or receipt of a positive SARS-CoV-2 test result, including 14 who had received an additional or booster dose; five of the 14 persons had received the additional dose <14 days before symptom onset. Six (14%) persons had a documented previous SARS-CoV-2 infection. The most commonly reported symptoms were cough, fatigue, and congestion or runny nose. One vaccinated patient was hospitalized for 2 days, and no deaths have been reported to date. Case investigations have identified exposures associated with international and domestic travel, large public events, and household transmission.

**TABLE:** Characteristics of reported confirmed B.1.1.529 (Omicron) variant SARS-CoV-2 cases (n = 43) — United States, December 1–8, 2021

Characteristic	No. (%)
**Age group, yrs**	
<18	4 (9)
18–39	25 (58)
40–64	10 (23)
≥65	4 (9)
**Sex**	
Male	17 (40)
Female	25 (58)
Unknown	1 (2)
International travel*	14 (33)
**COVID-19 vaccination status^†^**	
Unvaccinated	8 (19)
Partially vaccinated	0 (—)
Vaccinated	20 (47)
Vaccinated plus an additional dose^§^	14 (33)
Unknown	1 (2)
**Previous SARS-CoV-2 infection**	
Yes	6 (14)
No	21 (49)
Unknown	16 (37)
**Symptom profile**	
Symptomatic	40 (93)
Asymptomatic/Unknown	3 (7)
**Initial signs or symptoms** ^¶^	
Cough	33 (89)
Fatigue	24 (65)
Congestion or runny nose	22 (59)
Fever	14 (38)
Nausea or vomiting	8 (22)
Shortness of breath or difficulty breathing	6 (16)
Diarrhea	4 (11)
Loss of taste or smell	3 (8)
**Outcomes**	
Hospitalization	1 (2)
Death	0 (—)

* International travel within 14 days of symptom onset or, if asymptomatic, first positive SARS-CoV-2 test result.

^†^ An unvaccinated person had received no COVID-19 vaccine. A partially vaccinated person had received a COVID-19 vaccine but not completed the primary series ≥14 days before illness onset or receipt of a positive SARS-CoV-2 test result. A vaccinated person had completed the primary series of a Food and Drug Administration–authorized or approved COVID-19 vaccine ≥14 days before illness onset or receipt of a positive SARS-CoV-2 test result.

^§^ Among the 14 persons who were vaccinated and had received an additional dose, five had received the additional dose <14 days before symptom onset.

^¶^ Specific symptom information was available from 37 symptomatic persons.

## Measures to Slow Initial Travel-Related Spread of the Omicron Variant

On November 26, a Presidential Proclamation^§§^ suspended entry into the United States for noncitizens (as immigrants and nonimmigrants) who were present in any of eight countries in southern Africa (Botswana, Eswatini, Lesotho, Malawi, Mozambique, Namibia, South Africa, and Zimbabwe) during the 14 days preceding travel to the United States. This policy was intended to reduce overall travel volume from the region where Omicron was first identified to delay the introduction and spread of Omicron while U.S. public health measures were enhanced. Multiple factors were considered in determining the eight countries based on what was known about the spread of the Omicron variant at the time, including case counts, community transmission levels, and U.S.-bound travel volume from countries with cases. On December 2, CDC amended its existing Order requiring predeparture testing for all air passengers to the United States from any other country.^¶¶^ The Amended Order, effective December 6, shortened the window for obtaining a negative SARS-CoV-2 viral test result to no more than 1 day before the flight’s departure. A negative test result closer to the time of travel enhances reduction in transmission risk during travel (*6*).

On November 28, CDC expanded a voluntary airport-based genomic surveillance program in Atlanta, New York City, Newark, and San Francisco to prioritize recruitment of travelers from southern Africa for testing. The four participating airports receive a large, diverse volume of international travelers, including direct flights from southern Africa. Through this program, international air travelers are offered molecular testing of pooled samples collected upon arrival and a take-home collection kit (saliva collection for nucleic acid amplification test) to be used 3–5 days after arrival with subsequent sequencing of SARS-CoV-2–positive specimens; persons in pools with a positive test result are contacted and advised to get retested using the home collection kit or another method. Five pools collected November 30–December 6, representing 59 travelers, had evidence of SGTF. As of December 8, one of these pools was confirmed positive for Omicron, and four were pending. CDC continues to work with state and local health departments and other public health partners to conduct case investigation and contact tracing of travelers into and within the United States with confirmed COVID-19 attributed to the Omicron variant. As of November 8, all airlines are required to collect contact information for all inbound passengers to the United States to facilitate aircraft contact investigations and other follow-up of travelers when indicated.*** To date, at least one confirmed case attributed to the Omicron variant has been identified in the United States through these aircraft contact investigation efforts.

## Measures to Slow Domestic Spread of the Omicron Variant

CDC recommends prioritizing case investigation and contact tracing^†††^ for confirmed COVID-19 cases attributed to the Omicron variant. This prioritization should be balanced with maintaining case investigation and contact tracing for outbreaks of confirmed cases of SARS-CoV-2 infection in high-risk congregate settings (e.g., long-term care facilities, correctional facilities, and homeless shelters) and for persons at increased risk for severe COVID-19–related health outcomes. Timely case investigation and contact tracing can help ensure compliance with isolation and quarantine guidance^§§§^ and link persons with positive SARS-CoV-2 test results and their close contacts to testing and supportive services.

## Discussion

The first U.S. case of COVID-19 attributed to the Omicron variant was detected on December 1, 2021. Among the cases described in this report, the earliest report of symptom onset was November 15. For the week ending December 4, the Delta variant accounted for >99.9% of circulating SARS-CoV-2 variants. Given the 2–3 weeks from the time of specimen collection to availability of sequence data for analysis, it is likely that additional infections with Omicron from late November will be detected during the coming days. Scientists around the world are working to rapidly learn more about the Omicron variant to better understand how easily it might be transmitted and the effectiveness of current diagnostic tests, vaccines, and therapeutics against this variant. Many of the first reported cases of Omicron variant infection appear to be mild (*7*), although as with all variants, a lag exists between infection and more severe outcomes, and symptoms would be expected to be milder in vaccinated persons and those with previous SARS-CoV-2 infection than in unvaccinated persons. Characteristics of the cases described in this report might also not be generalizable because case findings might be associated with individual characteristics (e.g., persons with recent international travel might be more likely to be younger and vaccinated). Even if most infections are mild, a highly transmissible variant could result in enough cases to overwhelm health systems. The clinical severity of infection with the Omicron variant will become better understood as additional cases are identified and investigated. Scientists in South Africa and elsewhere have established systems that allow study of the laboratory, clinical, and epidemiologic characteristics; CDC is collaborating with health officials around the world to learn more about the characteristics of patients with Omicron variant infections.

The rapid emergence and worldwide detection of the SARS-CoV-2 Omicron variant underscores the importance of robust genomic surveillance systems and prompt information-sharing among global public health partners. During the past several years, CDC has intensified efforts to significantly expand genomic sequencing capacity at the federal and state levels. Through these investments, an average of 50,000–60,000 positive specimens are sequenced weekly as part of national SARS-CoV-2 genomic surveillance, which assisted with identifying initial cases of COVID-19 attributed to the Omicron variant in the United States.

A number of measures have been implemented throughout the COVID-19 pandemic to reduce the introduction and spread of SARS-CoV-2 in the United States through travel. For example, masks are required in indoor areas on public transportation conveyances traveling into, within, or out of the United States, and on the indoor premises of U.S. transportation hubs.^¶¶¶^ Current travel requirements and recommendations,**** surveillance programs, and efforts to educate travelers are intended to reduce COVID-19 transmission and support safer global travel. CDC is also supporting efforts to prevent, detect, and respond to COVID-19 internationally, including through support for laboratory and sequencing capacity and strengthening global vaccine programs.

Implementation of concurrent prevention strategies, including vaccination, masking, improving ventilation, testing, quarantine, and isolation, are recommended to slow transmission of SARS-CoV-2 and to protect against severe illness, hospitalization, and death from COVID-19. All persons aged ≥5 years should be vaccinated against COVID-19. Persons aged ≥18 years who completed a primary mRNA COVID-19 vaccination series ≥6 months previously or who received an initial Janssen (Johnson & Johnson) vaccine dose ≥2 months previously should receive a booster dose; persons aged 16–17 years are eligible to receive a Pfizer-BioNTech COVID-19 booster dose >6 months after completion of the primary series. Booster doses are especially urgent for those at higher risk of severe disease, such as persons residing in nursing homes and long-term care facilities. In addition, CDC recommends that everyone aged ≥2 years wear masks in public indoor places in areas of substantial or high transmission.

**Summary**

**What is already known about this topic?**

SARS-CoV-2 variant B.1.1.529 (Omicron), first reported to WHO on November 24, 2021, has been designated a variant of concern. Mutations in Omicron might increase transmissibility, confer resistance to therapeutics, or partially escape infection- or vaccine-induced immunity.

**What is added by this report?**

During December 1–8, 2021, 22 U.S. states reported at least one COVID-19 case attributed to the Omicron variant. Among 43 cases with initial follow-up, one hospitalization and no deaths were reported.

**What are the implications for public health practice?**

Implementation of concurrent prevention strategies, including vaccination, masking, improving ventilation, testing, quarantine, and isolation are recommended to slow transmission of SARS-CoV-2, including variants such as Omicron, to protect against severe illness and death from COVID-19.

## Footnotes

* SIG includes representatives from CDC, the National Institutes of Health (NIH), the Food and Drug Administration (FDA), the Biomedical Advanced Research and Development Authority (BARDA), the U.S. Department of Defense (DoD), the U.S. Department of Agriculture (USDA), and the U.S. Department of Health and Human Services (HHS). This interagency group focuses on the rapid characterization of emerging variants and actively monitors their potential impact on critical SARS-CoV-2 countermeasures, including vaccines, therapeutics, and diagnostics.

†
https://www.gisaid.org

§
https://www.ncbi.nlm.nih.gov/sars-cov-2

¶
https://www.cdc.gov/coronavirus/2019-ncov/variants/spheres.html

**
https://covid.cdc.gov/covid-data-tracker/#variant-proportion

†† 45 C.F.R. part 46.102(l)(2); 21 C.F.R. part 56; 42 U.S.C. Sect. 241(d); 5 U.S.C. Sect. 552a; 44 U.S.C. Sect. 3501 et seq.

§§
https://www.whitehouse.gov/briefing-room/presidential-actions/2021/11/26/a-proclamation-on-suspension-of-entry-as-immigrants-and-nonimmigrants-of-certain-additional-persons-who-pose-a-risk-of-transmitting-coronavirus-disease-2019

¶¶
https://www.cdc.gov/quarantine/fr-proof-negative-test.html

***
https://www.cdc.gov/quarantine/order-collect-contact-info.html

†††
https://www.cdc.gov/coronavirus/2019-ncov/php/contact-tracing/contact-tracing-plan/overview.html

§§§
https://www.cdc.gov/coronavirus/2019-ncov/your-health/quarantine-isolation.html

¶¶¶
https://www.cdc.gov/quarantine/masks/mask-travel-guidance.html

****
https://www.cdc.gov/coronavirus/2019-ncov/travelers/international-travel/index.html
